# History of an Operation for Artificial Anus, Successfully Performed by a New Method in a Case of Congenital Absence of the Rectum

**Published:** 1844-10

**Authors:** 


### ART. XIV.

1. *Histoire d'une Opération d'Anus artificiel pratiqué avec succès par un nouveau Procédé, dans un cas d'Absence congéniale de l'Anus; suivie de quelques réflexions sur les Obturations du Rectum.* Par J. Z. AMUSSAT. *Lu à l'Académie des Sciences, le* 2e *Nov.* 1835. (*Gazette Médicale de Paris,* 28*th Nov.* 1835.)

*History of an Operation for Artificial Anus, successfully performed by a New Method in a case of congenital absence of the Rectum.* By J. Z. AMUSSAT. *Read before the Academy of Sciences,* 2d *Nov.* 1835.

2. *Mémoire sur la Possibilité d'établir un Anus artificiel dans la Région lombaire sans pénétrer dans le Péritoine. Lu à l'Académie Royale de Médecine, le* 1er *Oct.* 1839. Par J. Z. AMUSSAT.—*Paris,* 1839.

*Memoir on the Possibility of establishing an Artificial Anus in the Lumbar Region without opening the Peritoneum. Read before the Royal Academy of Medicine,* 1st *Oct.* 1839. By J. Z. AMUSSAT.—*Paris,* 1839. 8vo, pp. 210.

3. *Deuxième Mémoire sur la Possibilité d'établir un Anus artificiel dans les Régions lombaires sans ouvrir le Péritoine.* Par J. Z. AMUSSAT. *Lu à l'Académie Royale de Médecine,* 6 *Sept.* 1841.—*Paris,* 1841.

*Second Memoir on the Possibility of establishing an Artificial Anus in the Lumbar Regions without opening the Peritoneum.* By J. Z. AMUSSAT. *Read before the Royal Academy of Medicine,* 6*th Sept.* 1841.—*Paris,* 1841. 8vo, pp. 60.

4. *Troisième Mémoire sur la Possibilité d'établir une ouverture artificielle sur le Colon lombaire gauche sans ouvrir le Péritoine, chez les Enfans imperforés. Lu à l'Académie Royale des Sciences, le* 4 *Juillet,* 1842. Par M. AMUSSAT. (*L'Examinateur Médicale,* Nos. 16, 17, 18; Ann. 1843.)

*Third Memoir on the Possibility of establishing an Artificial Opening in the left lumbar Colon without opening the Peritoneum in Infants born with imperforate Anus. Read before the Royal Academy of Sciences,* 4*th July,* 1842. By M. AMUSSAT.

5. *Du Cancer du Rectum et des Opérations qu'il peut réclamer, parallèle des Méthodes de Littré et de Callisen pour l'Anus artificiel.* Par A. VIDAL (DE CASSIS.)—*Paris,* 1842.

*On Cancer of the Rectum and the Operations it may require; with a parallel between the Methods of Littré and of Callisen, for establishing an Artificial Anus.* By A. VIDAL (DE CASSIS.)—*Paris,* 1842. 8vo, pp. 128.

6. *A Case of Carcinomatous Stricture of the Rectum, in which the descending Colon was opened in the Loin.* By ALFRED JUKES.—*London,* 1842. 4to, pp. 24. With Plates.

THE formation of an artificial anus may be obviously requisite, or more or less doubtfully indicated, under a considerable variety of circumstances; but notwithstanding that the subject is of necessity touched on in almost every systematic work on surgery, and has been also considered in several detached papers and memoirs, and in some special treatises on the surgical diseases of the intestinal canal, the propriety of the operation and the most eligible mode of performing it in certain cases have been but little discussed. The object of M. Amussat's memoirs is to establish settled rules of practice wherever vagueness and uncertainty have hitherto existed, and also to substitute improved methods of operating for those hitherto in use. We shall first consider M. Amussat's views respecting the formation of an artificial anus in the anal region, and then examine the memoirs on the performance of the operation on the lumbar colon.

The malformations commonly termed imperforate anus may be divided into two classes. In one the gut is not impervious, but opens in some preternatural situation, as into the bladder, the urethra, or the vagina. In the other the rectum is really imperforate, being either obliterated to a greater or less extent, or obstructed by a membranous septum, at a variable height above a naturally formed anus; or else it terminates in a cul de sac at an indeterminate height in the pelvis without any external aperture. The operation hitherto performed in all those cases is, M. Amussat maintains, quite insufficient, and ultimately attended with uniform failure. In the first two cases of imperforate anus on which M. Amussat operated, the rectum terminated between one and a half and two inches from the surface; in each case he formed an artificial anus by the usual method of simply cutting down on the rectum, but both infants died jaundiced in a few days, which he attributed to absorption of the bile, and meconium consequent on their coming into contact with a wound of such considerable extent; he therefore determined in future to adopt a method calculated to obviate that inconvenience, which we shall perhaps best explain by giving an abstract of the case in which he first carried it into effect.

A female infant was born with the following malformation: The vulva and anus were both naturally formed externally, but the recto-vaginal septum was deficient above, and only existed inferiorly to the extent of about one third of an inch, so that the finger could be passed from either canal into the other. The upper portion of the rectum had no communication with the cloaca common to the vagina and the anal portion of the rectum; but its closed extremity could be felt at a height of about two inches towards the left sacro-sciatic angle. The anus thus communicated directly with the vagina above the imperfect septum already mentioned, but had *no connexion* with the rectum, which terminated two inches above it, and was in fact, properly speaking, *deficient* to that extent. Under these circumstances M. Amussat determined

"To make an incision anterior to the coccyx but posterior to and not involving the vaginal anus, to detach the posterior wall of the vagina from the coccyx and sacrum with the finger or the knife, to reach the cul de sac of the rectum, seize it with a hook, detach its entire circumference rather with the finger than by the knife, draw it down to the external wound, open it freely, give exit to the meconium, and secure, by points of interrupted suture, the edges of the opening in the intestine to the lips of the cutaneous wound." (Gaz. Méd. de Paris, 1835, p. 755.)

The operation thus planned was executed with much less difficulty than had been anticipated, and certainly with a most satisfactory result, for the last notice we find of the case is eight and a half years after the performance of the operation, and the patient then enjoyed excellent health. Nothing is said as to how far she possessed command over the retention and evacuation of the feces, and the artificial anus, it would appear, retained a tendency to contract, which commenced twelve days after its formation, as the dilatation of the rectum was maintained by the occasional introduction of a cylinder of wood. (Gaz. Méd. de Paris, 1843, p. 100; Gaz. des Hôp. de Paris, 1843, p. 67.)

The essential feature in the operation performed in this case is the bringing down the upper portion of the rectum to the level of the external wound, and thus obtaining along the entire extent of the artificial trajet the presence of tissues whose organization is adapted to the functions they are called on to discharge, and its execution "is based on the possibility of elongating the extremity of the great intestine from one to two inches, the inferior mesenteric artery alone preventing a still greater elongation, which the sigmoid flexure of the colon would readily admit." But in order to ensure a sufficient elongation of the gut, M. Amussat, in his third memoir, especially insists on the necessity of forming the artificial anus, not in the situation of the natural aperture, but posterior thereto, in the coccygeal region. When the case we have just given an abstract of was communicated to the Royal Academy of Medicine, it was suggested that the rectum should have been connected with the anus in order to take advantage of the presence of the sphincter; and M. Amussat was then inclined to admit the propriety of the suggestion, and regretted that the apprehension of forming a recto-vaginal fistula had deterred him from making the attempt; he subsequently, however, became convinced that the rectum probably could not have been sufficiently elongated for that purpose, and in a similar, or indeed in any other case, would again form the anus in the region of the coccyx, because the rectum has a shorter distance to traverse to reach the surface if drawn directly *backwards*, than if drawn downwards to the situation naturally occupied by the anus; he therefore makes the external incision immediately in front of or even on the left side of the os coccygis, which bone he would excise, if necessary, to gain room; and in one case did remove its extremity for that purpose. (L'Examinateur Méd. 1843, pp. 198-216.)

The cases in which M. Amussat recommends this operation include almost every variety of malformation of the rectum. He would apply it in every case of true imperforation of the rectum, in which it was possible to reach the gut, with the exception of those only in which the anus, otherwise well formed, is obstructed by a mere *superficial* membrane; but if the septum, however thin and yielding, however it may be distended by the accumulation of meconium, is situated *above* the anus, he insists that it is insufficient to destroy the septum, "that no case can be cited in favour of that method," which fails because of the difficulty of keeping an opening above the anus dilated; and M. Amussat therefore lays it down as a rule, that in such cases "we should operate as if there was no anus, as if the rectum was completely deficient throughout the entire extent of its anal extremity, and cut backwards and draw the rectum not downwards to the anus but directly backwards," etc. (L'Examinateur Méd. 1843, p. 198.) When the rectum opens into a neighbouring organ, the indication, according to M. Amussat, is the same as in complete imperforation of the anus, unless the abnormal anus sufficiently discharges the function of defecation; but when the gut communicates with the bladder or with the urethra, its posterior wall only should be drawn down. In those rare cases where the intestine opens on the parietes of the abdomen, the operation is inapplicable, and if the orifice is insufficient and cannot be dilated, an artificial anus should be formed in the lumbar region.

M. Amussat, we have seen, attaches no importance to bringing the rectum into connexion with the sphincter ani; on the contrary, he deprecates the attempt to do so for the reasons already assigned. We shall hereafter see that M. Amussat, frequently and explicitly as he expresses this opinion, is somewhat inconsistent with himself on this point; but for the present we shall only observe that when the anus exists we think it of great importance to preserve it; and the rectum, *if* it is to be drawn down at all, should, if possible, be brought into communication with it. When there is no trace of the anus externally, does the sphincter exist, and if so, can its functions be preserved to the artificial opening? On this subject there is a strange diversity of statement. J. L. Petit (Mém. de l'Acad. de Chirurg.) says that in imperforate anus the sphincter indeed exists, but is so contracted, wasted, and confounded with the surrounding parts, that it is difficult or rather impossible for it to resume its functions with whatever care the operation may be performed. P. F. Blandin (Dict. de Méd. et Chirurg. Prat.) says, "When the anus is completely absent, I have ascertained that the sphincter is absent also, whether the skin does or does not present an indication of the natural situation of the rectum;" and this very respectable authority in surgery actually thence concludes, that as an artificial anus established in the perineum must occasion incontinence of feces in a very inconvenient situation, we should prefer opening the sigmoid flexure of the colon whenever the anus is truly imperforate. Velpeau is also of opinion that the sphincter is always absent in such cases. On the other hand, M. Roux of Brignolles (Mém. de l'Acad. Royale de Méd. 1835, t. iv, p. 183) maintains that imperforate anus, with the deficiency of the lower portion of the rectum, is occasioned by the deviation or the insufficient size of the branch of the pubic artery by which that part of the rectum is supplied, but that the levator ani and sphincter muscles, being supplied from the ischiatic artery, exist independently of the rectum, and are invariably present, and that, consequently, the operation should be so conducted as to preserve them in connexion with the newly-formed anus, and thus enable the patient to have command over the retention of the feces. M. Goyraud (Journ. Hebdom. 1834, t. iii, p. 245) again lays it down as an undeviating rule, that the deeper portion of the sphincter ani is always absent when the lower portion of the rectum is deficient, but that the superficial portion of the muscle not only always exists, but is preternaturally developed in those cases, and he thence comes to the same practical conclusion as M. Roux. The truth of course lies between those general allegations. M. Blandin's and M. Velpeau's dissections prove that the sphincter is sometimes absent; while the experience of MM. Roux and Goyraud show that it is sometimes present; but it remains to be deduced from facts how far the presence or rather the preservation of the sphincter, in connexion with the artificial anus, is essential to prevent incontinence of feces. On the one hand, in MM. Roux and Goyraud's cases, the anus was formed with the greatest care in the mesial line of the sphincter muscle; in the former case the child lived ten days, during which time the anus seemed to be under the influence of the muscle, and even enemata were retained; and in the latter case, the anus, we are told, fulfilled all the functions of a natural anus six weeks after the operation, which is the latest report we have of the case. On the other hand, B. Bell, in his 'System of Surgery,' mentions two cases "in which the gut lay deep," on which he operated, *probably* without preserving the sphincter, as he "cut backwards along the coccyx;" but in both he "was fortunate enough to form an anus which for a good many years has continued to answer the purpose *sufficiently* well;" indeed the difficulty of keeping the passage open is the only circumstance of which Bell complains in those cases. A most valuable case is recorded by Mr. Ferguson, (Edinb. Med. and Surg. Journal, 1831, p. 363; and 'A System of Pract. Surgery,' p. 527,) in which the rectum terminated in the membranous portion of the urethra, and an incision having been made into the gut, we presume, (but, as was supposed at the time, into the neck of the bladder,) the boy lived and throve; and what is very important, possessed command over the evacuation of both urine and feces till he was six years old. As Mr. Ferguson, after a post-mortem examination, expresses a doubt whether the sphincter and levator ani muscles existed, it may be at the least inferred that they were not in a condition to have exerted any efficient control over the function of defecation. M. Amussat, from the fact that patients after excision of the lower extremity of the rectum have retained control over the alvine evacuations, thinks the same power would probably exist after his operation for artificial anus, the more so, as he is of opinion, that the interior outlet of the abdomen is so disposed, independently of its muscular apparatus, as to favour voluntary retention of the feces. It would be satisfactory to know how far his patient enjoyed that power; but this unfortunately is not stated: the inconvenience in this respect, however, if any, would appear to be but slight, for as in Bell's cases, the only annoyance mentioned is the necessity of keeping the anus mechanically dilated. Though we must confess that the few facts we are able here to bring together are insufficient to determine the question we have put, they yet seem to justify the hope, that whether we operate for imperforate anus by the old method or that of M. Amussat, the power of retaining the feces may be preserved, or that, at all events, the infirmity will be greatly less than M. Blandin and others anticipate as inevitable.

It cannot, we conceive, be questioned that the method adopted by M. Amussat in his first case, was a most meritorious and happy improvement of the older operation for forming an artificial anus. The intestine having been within reach of the finger could have been easily opened, (though perhaps not very easily kept pervious,) but from the nature of the malformation in no other way than by the novel method of bringing down the cul de sac of the rectum to the level of the integuments, could an infirmity of the most disgusting kind have been obviated. But though in this and analogous cases M. Amussat's operation is a most admirable improvement, we think he seeks to extend the method too far.

In the first place M. Amussat unquestionably exaggerates the imperfection and failure of the ordinary mode of operating in imperforation of the rectum and of the anus, when he says in some passages that "every," and in others that "almost every" operation has failed in which more than a superficial membrane had to be divided, and even alleges, as we have already observed, that there is no example of the division of a simple septum within the rectum having proved successful. That M. Amussat has fallen into a strange mistake in thus imputing uniform failure to the operation by simple incision sufficiently appears from the cases of B. Bell and Mr. Ferguson, which we have already referred to for another purpose, and we need not occupy space by multiplying quotations to the same effect, but so thoroughly persuaded is M. Amussat that such is the case, that he contrasts this supposed uniform failure with the few cases in which Littré's operation has succeeded, and seems to infer that the latter is the preferable operation, (Gaz. Méd. de Paris, 1835, p. 758.) But though we thought it right thus to notice the inaccuracy of M. Amussat's most cogent argument, (most cogent if true,) for general adoption of his new method, we shall not further enter on the discussion of the various objections which we think might be urged against its application in various cases. We could only suggest theoretical objections, and we prefer making M. Amussat criticise himself, and shall show from his own words that he distrusts his own rules, and also directly violates in practice the precepts which he inculcates as undeviating canons in the didactic parts of his memoirs, that part, we may observe by the way, which many, perhaps most, readers would be the more likely to consult. In the memoir in the 'Examinateur Méd.' 1843, he republishes the case of which we have given an abstract, and terminates it by a caution not appended to the case as originally printed, and moreover, not hinted in the rules for operating laid down in the first part of this *very same* memoir, where it would certainly have been much better placed than as an incidental observation in the report of a case. The caution in question is, "it is in general not right to persevere and count too much on the possibility of depressing the rectum, lest serious or even fatal consequences might result," (Loc. cit. p. 215 ;) and moreover, we find that in four other cases published in this very memoir, M. Amussat adopted his own method, (which we are told a few pages previously is universally applicable,) in *one only*. Of those four cases, one (second in the memoir) cannot be taken into account, as M. Amussat failed to reach the rectum, though after death the incision was found to have so nearly attained its extremity, that had he persevered, (which the exhausted condition of the patient forbad,) he would probably have achieved his object. In the second case, (third in the memoir,) though the rectum lay at the depth of one inch from the surface, the improved operation was *not* performed, but the gut was opened by a simple incision. The child died two days after. In the third case, (fourth in the memoir,) M. Amussat made a deep incision in the coccygeal region after having excised the extremity of the os coccygis to gain room without discovering the rectum; he then proposed to open the lumbar colon, but the child's parents having refused their consent, he resumed the original operation, found the rectum attached to the upper part of the vagina, and having drawn it down, secured it to the lips of the external wound. Death occurred here also in two days. In the last case, (fifth in the memoir,) there was a well-formed anus, (in a male child,) about three quarters of an inch above which the rectum was obstructed by a septum through which no fluctuation, nothing indicating the existence of the upper portion of the rectum could be felt; behind the anus, however, fluctuation could be detected especially when the child struggled, or when the abdomen was compressed. A deep incision in this situation gave exit to meconium, and the membrane separating the two portions of the rectum (posteriorly,) was then cut away so that the anus and wound formed but one opening. Two months subsequently the child was in perfect health. In some reflections on this case M. Amussat observes, that it is important as regards diagnosis, for when no fluctuation could be felt by the finger passed into the rectum, we would scarcely anticipate detecting it lower down behind the anus, but would rather expect that the other extremity of the intestine was situated high in the pelvis. The circumstance, however, is easily explained by admitting that the upper portion of the rectum being more and more distended with meconium and constantly impelled downwards by the efforts of the infant, descended sufficiently low to be recognized externally. J. L. Petit relates a case which shows that this is the true explanation. A surgeon failed to attain the rectum through an incision in the perineum, but another surgeon three hours subsequently found the closed extremity of the rectum distended with meconium protruding through the wound ; and hence the important rule of practice that when the rectum is not discovered in the first instance, we should before resorting to Littré's or Callisen's operation, if the condition of the child admits of delay, wait to see if the rectum shall protrude towards the wound, in which direction it will probably experience least resistance. But to return from this digression : M. Amussat there formally contradicts the rules of practice laid down without any reserve or limitation, a few pages before in this very same memoir, for he says,

"Previous to this case I always endeavoured to bring down the upper portion of the rectum to the anus, after having incised the lower portion backwards. But obviously the operation performed in this case is much simpler and greatly preferable. It resembles an extensive operation for fistula in ano." (L'Expérience, 1843, p. 217.)

On M. Amussat's own showing then, the operation which, as we have seen, he recommends as universally applicable, must be performed with due caution and reserve, in fact only in exceptional cases, for when the cul de sac of the rectum lies high in the pelvis, we must not " persevere and count too much on the possibility of depressing the rectum, lest serious or even fatal consequences might result ;" and in two of the cases above referred to, he considered his new operation unnecessary in one, and improper in the other. Although then M. Amussat's operation may be a most useful modification of the ordinary proceeding in some cases, it certainly is not essential to success to adopt it universally, and if it were so, M. Amussat acknowledges that it might be impracticable when the rectum lay at any depth from the surface, that is to say in the very cases in which on his own principles, it would be most necessary.

We may here observe that M. Amussat, and also MM. Roux and

Goyraud, blame their predecessors for not having sought for the rectum with sufficient boldness, and claim no small merit for introducing as an important novelty, free incisions with the knife guided by the finger in lieu of punctures with a trocar or lancet. We freely admit that the former is decidedly the preferable method, but those gentlemen are greatly mistaken in supposing that it remained for them to suggest. B. Bell long since practised and taught this supposed novelty, which has since received the sanction of numerous authorities down to the present day.

When every effort to attain the rectum from below proves ineffectual, M. Amussat asks, should we endeavour to open the cul de sac of the peritoneum, and seize whatever intestine may present itself, at the risk of drawing down a portion of the small intestine? and to this question he replies in the affirmative! (L'Examinat. Méd. 1843, p. 198.) We here, however, have only another example of our author inculcating a rule from which he shrinks in practice, for we need hardly say that in none of his cases did he act on the precept which he here lays down as his deliberate and unqualified opinion. Indeed M. Amussat's opinions are very unsettled and fluctuating; in the 'Bulletin de l'Acad. Royal de Méd.' (Juin 1842, No. xviii, p. 802,) we find him, in contradiction to the entire tenor of his memoirs both of antecedent and subsequent date, expressing his opinion,

"That, in cases of imperforate anus, we should not try to reach the intestine from the perineal or coccygeal regions, except when fluctuation is manifestly perceptible in those regions. If fluctuation cannot be detected, we expose ourselves to the risk of performing an useless operation, after which it would be impossible to attempt, with any chance of success, the formation of an artificial anus in the left lumbar region; for the infant exhausted by pain and loss of blood, would rarely endure the second operation with impunity."

Now, not to mention the passage referred to immediately before this last quotation, in which he recommends the extreme and extravagant proceeding of drawing down a portion of small intestine through the pelvic cul de sac of the peritoneum, M. Amussat, in numerous other passages, says, that the operation in the lumbar colon should never be undertaken until we have in vain attempted to reach the rectum from below. And in contrast with the unqualified statement in the last quotation, that the existence or non-existence of fluctuation is to govern our proceedings, we shall give the following extract from 'L'Examinateur Méd.,' 1843, p. 198:

"But in fine, in what cases should we operate above, that is to say in the abdomen, or in the lumbar region? It is very difficult to answer this question at present, as the problem is not yet solved, and cannot be so save by the results of attempts repeated from below."

We might accumulate similar examples of self-contradiction on the part of M. Amussat, but enough, perhaps more than enough, has been done in this respect, and the last quotations naturally lead us to the consideration of the operations in the abdominal and lumbar regions.

Without referring to what is said respecting the ancients, especially Praxagoras, M. Littré was the first in modern times at least to suggest the practice of forming an artificial anus in the abdomen in cases of imperforated rectum, otherwise irremediable; his ideas are stated very generally, for it is expressly said that the details are purposely suppressed, inasmuch as any dexterous surgeon may easily imagine them; it is generally said, however, that Littré directs us to cut into the cavity of the peritoneum in the left iliac region, open the sigmoid flexure of the colon, and secure the open intestine in contact with the wound by means of a thread passed through the mesentery. It is very strange that this interpretation of Littré's proposal, though adopted by every writer with whom we are acquainted, including M. Amussat, is quite incorrect. Littré's proposition is contained in the following few words: "Il faudrait faire un incision au ventre, *et recoudre ensemble les deux parties* apres *les* avoir rouvertes, ou *du moins* faire venir *la partie supérieure de l'intestine*, a la plaie du ventre, que l'on ne refermemerait jamais, et qui ferait la fonction d'anus." (Hist. de l'Acad. des Sciences, 1710, p. 36.) And thus it appears that Littré's leading idea was the possibility of uniting the upper and lower portions of the rectum by suture after having opened their closed extremities, (for in the case whch gave rise to his suggestion the rectum was pervious both above and below an obstruction about an inch in length,) and this failing, we should *at least* connect the upper portion of the *rectum* with the external wound. There is not a word about the sigmoid flexure of the colon, the thread in the mesentery, &c. The idea of forming an artificial anus in the abdomen, however, is here very clearly suggested, but appears to have been first acted on in 1776, by Pillore, of Rouen, but under very different circumstances from those contemplated by Littré. Pillore's patient was an adult whose rectum was completely obstructed by a cancerous tumour; and the artificial anus was formed in the *cæcum*, which was opened through the peritoneum: the patient survived twenty-eight days, and the immediate cause of his death seems to have been intense inflammation of the jejunum, caused by two pounds of metallic mercury administered a month previous to the operation, and which had lodged in and displaced that portion of the small intestine. A. Dubois, in 1783, was the first who performed the operation on an imperforate infant, but no particulars of the case are known, save that the child died on the tenth day. Duret, in 1793, comes next in order, and his case is very remarkable: 1st. Because he previously tried Callisen's operation on the dead body of a child fifteen days old; but finding that he had wounded the peritoneum, as the descending colon was attached to the mesentery, he abandoned this operation and performed that of Littré. 2d. Because the operation was completely successful, the patient having certainly survived forty-two years; and we conclude from M. Amussat's first memoir, p. 187, was still alive in 1839.

We cannot notice the succeeding cases in detail, but shall throw into a tabular form the more important particulars of all those that are known, which we borrow from M. Amussat's 'Memoirs,' except where another reference is specified; and although it is anticipating somewhat, we shall here insert tables of the known cases of Callisen's operation, as modified by M. Amussat, as it will be convenient to place the results of the two operations in contrast in a compendious form.

### Table I.

**Cases in which Littré's operation for establishing an artificial anus was performed for malformation of the rectum.**

| Operator, and date of operation. | Sex. | Age. | Malformation. | Situation of artificial anus. | Result. |
|---|---|---|---|---|---|
| 1. Dubois. 1783 | | 3 days | Imperforate anus | | Death in 10 days |
| 2. Duret.* 1793 | Boy | 2 days | Do. | Sigmoid flexure of the colon | Living and enjoying good health in 1835, being then 42 years of age. |
| 3. Desault. 1794 | Do. | Do. | Do. | Do. | Death in 4 days. |
| 4. Voisin. 1798 | | | Do. | | Unknown. |
| 5. Desgranges 1800 | Girl | 4 years | Rectum opened into vagina | Supposed to be in sigmoid flexure of the colon | In good health 8 months after the operation. |
| 6. Voisin. 1802 | Boy | 10 days | Total absence of large intestines. Ilium terminated in a small fecal fistula in hypogastrium | Ileum | Death in 4 days. |
| 7. Duret.* 1809 | Do. | 2 days | Imperforate anus | Sigmoid flexure of the colon | Death 4th or 5th day. |
| 8. Legris. 1813 | | | Rectum opened into urethra | Supposed to be sigmoid flexure of the colon | Death in 17 days. |
| 9. A surgeon of Lyons. | | | Imperforate anus | Sigmoid flexure of the colon | Successful. |
| 10. A surgeon of Brest. | Girl | | Do. | Do. The ovary was included in the ligature passed through the mesentery | Death. |
| 11. Serrand. 1813 | Do. | 60 hours | Rectum imperforate 1½ inch above anus | Sigmoid flexure of the colon | In good health 22 months after. |
| 12. Freer.* 1816 | | | Imperforate anus | Do. | Death in 3 weeks. |
| 13. Miriel. 1816 | Girl | 2 days | Rectum imperforate ½ inch above anus | Do. | Living and enjoying excellent health in 1835, being then aged 19. |
| 14. Ouvrard. 1820 | Do. | Do. | Imperforate anus | Supposed to be sigmoid flexure of the colon | Unknown. |
| 15. Miriel.* 1822 | Boy | 84 hours | Rectum imperforate 1¼ inch above anus | Sigmoid flexure of the colon | Living 13 months after operation. |
| 16. Do. 1823 | Do. | 6 days | Imperforate anus | Do. | Lived 27 months. |
| 17. Bizet.* 1830 | Do. | | Do. | Do. | Lived 1 month. |
| 18. Do. | | 100 hours | Do. | Do. | Death next day. |
| 19. Klewig.* 1835 | Boy | 3½ days | Do. | Do. | Lived 3 years. |
| 20. Roux. | | | Do. | Do. | Death in 2 hours. |
| 21. Bougon.† 1838 | | 13 days | Do. | Descending colon in left lumbar region | Death in 28 hours. |

* In these cases an attempt was made, in the first instance, to establish an artificial anus in the anal region.

† In this case M. Bougon intended to perform Callisen's operation; but as the cavity of the peritoneum was opened, the case is properly to be classed here. (Gaz. des Hôp. de Paris, 1838, p. 78.)

### TABLE II.

Cases in which Littré's operation was performed on the adult, in consequence of obstruction of the intestinal canal.

| Operator, and date of operation. | Sex. | Age. | Disease. | Situation of artificial anus. | Result. |
|---|---|---|---|---|---|
| 1. Pillore. 1776 | Man | | Cancer of the rectum | Cecum | Death 28th day. |
| 2. Fine.* 1797 | Woman | 63 years | Do. | Transverse colon | Lived upwards of 3½ months. |
| 3. Freer. 1818 | Man | 47 years | Stricture of rectum | Sigmoid flexure of the colon | Death 8th day. |
| 4. Pring. 1820 | Woman | 64 years | Do. | Do. | Alive 6 months after the operation. |
| 5. Martland. 1824 | Man | 44 years | Do. | Descending colon | Alive 17 years after the operation. |
| 6. Velpeau. 1839 | Woman | | Do. | Sigmoid flexure of the colon | Death in 2 days. |
| 7. Amussat. 1840 | Do. | 47 years | Do. | Do. | Death in 24 hrs. |
| 8. Thierry.† 1840 | Man | | Cancer of sigmoid flexure of the colon | Commencement of ascending colon in right iliac fossa | Death in 28 hrs. |

* M. Fine intended to establish the artificial anus in the small intestine, but the transverse colon presented and was opened.

† M. Thierry intended to establish the artificial anus in the cecum, without wounding the peritoneum; but the cavity of the abdomen was opened, and the anus formed in the colon, which intestine lay in front of the cecum, having been displaced by the lodgment of metallic mercury, administered to overcome the obstruction. (L'Expérience; 6th Oct. 1842, p. 209.)

---

### TABLE III.

Cases in which Callisen's operation, modified by M. Amussat, was performed for malformation of the rectum.

| Operator, and date of operation. | Sex. | Age. | Malformation. | Result. |
|---|---|---|---|---|
| 1. Amussat. 1842 | Boy | 2 days | Rectum imperforate 1½ inch above anus | Living upwards of a year after.* |
| 2. Do. 1842 | Do. | Do. | Rectum imperforate 1 inch above anus | Death 7th or 8th day. |
| 3. Do. 1843 | Do. | Do. | Imperforate anus | Alive and in good health 7 weeks after.* |
| † | Do. | | | Attained adult age. |
| Dupuytren.‡ | | | Imperforate anus | Death from peritonitis. |

* Gaz. Méd. de Paris, 1842, p. 267; and 1843, p. 99. Gaz. des Hôp. de Paris, 1843, p. 67. L'Examinateur Méd., 1843, pp. 229-32.

† The reference to this case in MM. Roche and Sanson's work has been already given. The authenticity of the case is doubtful, and M. Amussat's modification was not, it is to be presumed, practised, as the date of the case, if genuine, is long anterior to his memoirs.

‡ This case is briefly mentioned in the 'Leçons Orales.' The artificial anus was formed in the *cecum*, without wounding the peritoneum; and although it consequently does not strictly come under the head of Callisen's operation, yet, as the essential difference between Callisen's and Littré's operation is, that the peritoneum is opened in the latter and not in the former, the case seems to be most properly introduced here. In all M. Amussat's cases the artificial anus was established in the descending colon.

### TABLE IV.

**Cases in which Callisen's operation, modified by M. Amussat, was performed on the adult, in consequence of obstruction of the intestinal canal.**

| Operator, and date of operation. | | Sex. | Age. | Disease. | Situation of artificial anus. | Result. |
|---|---|---|---|---|---|---|
| 1. Amussat | 1839 | Woman | 48 years | Cancer of the rectum | Descending colon | Lived 5 months. |
| 2. Do. | 1839 | Man | 62 years | Do. | Do. | Alive 2 years and 3 months after the operation. |
| 3. Do. | 1841 | Woman | 50 years | Situation and nature of obstruction unknown | *Ascending* colon | Alive and healthy much improved 2 months after the operation. Feces passed several times by anus. |
| 4. Do. | 1841 | Do. | 60 years | Cancer of the rectum | Do. | Death 10th day. |
| 5. Do.* | 1841 | Man | 57 years | Incomplete obstruction from a tumour in left iliac fossa | Do. | Alive 75th day. Health much improved. |
| 6. Baudens.† | 1841 | Woman | | Stricture of rectum: not malignant | Do. | Death 5th day. |
| 7. Clements.‡ | 1841 | Do. | | Situation and nature of obstruction unknown at time of operation | Do. | Recovery. At 7th week a quantity of plumstones discharged from bowels. |
| 8. Teale.§ | 1842 | Do. | 54 years | Stricture of sigmoid flexure of the colon | Descending colon | Death 7th day. |
| 9. Jukes. | 1842 | Do. | 30 years | Cancer of the rectum | Do. | Death 16th day. |
| 10. Amussat. | 1844 | Woman | 53 years | Situation and nature of obstruction unknown | Descending colon | Patient alive and health much improved 1 month after operation. |

The operation, commonly termed Callisen's operation, consists in opening the descending colon from the lumbar region without wounding the peritoneum. M. Amussat, besides modifying the steps of the operation, has also extended it to the ascending colon, in which he has been followed by some other practitioners. It is not known who first suggested this proceeding. All that Callisen says is as follows:

"Si cavum intestinale cultro vel paracentesio attingi nequeat, vix servari poterit æger. Que proposita sub hoc rerum statu fuit incisio intestini cæci vel coli descendentis, sectione in regione lumbari sinistra ad marginem musculi quadrati lumborum facta, ut anus paretur artificialis, remedium præbet omnino incertum, atque hac operatione vix vita miselli servari poterit. Quanquam intestinum in hoc loco facilius attingatur, quam supra regionem inguinalem." (Systema Chirurg. Hodiern. Hafniæ, 1817, t. ii, p. 842.)

* Gaz. des Hôp. de Paris, 1842. Mars 26, No. 27.
† Gaz. des Hôp. de Paris, 1842. Avril 19, No. 47.
‡ A case of carcinomatous stricture of the rectum, &c., by A. Jukes, p. 16.
§ Prov. Med. and Surg. Journ., March 19th, 1842, p. 486.
|| Gaz. des Hôp. de Paris, July, 1844, p. 356.

Callisen mentions, then, the operation as having been proposed; he does not say by whom. He neither claims it as his own, nor does he allude to the distinguishing feature of not opening the peritoneum. All that is as yet further known respecting the early history of this operation is, 1st. That Sabatier, *without, however, giving any reference or authority*, says, (Méd. Opérat., 2e ed. t. iii, p. 337,) that Callisen tested the practicability of opening the left lumbar colon without wounding the peritoneum, on the body of a child who died with imperforate anus; that in his first attempt, he opened the peritoneum, but that, by a second incision further back, he attained his object as he had proposed; in effecting which, however, he acknowledged he was much aided by passing his fingers into the first incision, and thus fixing the intestine. 2d. That Duret, as we have already mentioned, made a similarly unsuccessful attempt on the dead subject. 3d. That MM. Roche and Sanson (Nouv. Elem. de Path. Medico-Chirurg., 3d ed. t. v, p. 440) say, a young man had been pointed out to them on whom Callisen's operation had been successfully performed immediately after birth; but that they could neither ascertain the particulars of the operation nor permission to examine the parts. 4th. That M. Bougon (Gazette des Hôp. de Paris, 1838, p. 78) attempted the operation on an imperforate infant, but failed, inasmuch as he opened the peritoneum, as was ascertained by dissection, the child having died. And it may not be out of place to mention, 5th, That Dupuytren unsuccessfully opened the cecum without wounding the peritoneum, in a case of imperforate anus. These, we believe, are the only known facts, and the whole stream of authorities, from Callisen down to the period when M. Amussat resumed the investigation of the subject, condemned Callisen's operation, some maintaining that its performance was impossible, others that it was incomparably more difficult and not less dangerous than Littré's operation. Allan alone (Recueil Périod. de la Soc. de Méd. de Paris, t. i, p. 123) expressed an opinion that Callisen's method was deserving of further consideration. We must refer to M. Amussat's memoirs for the specific quotations which show that Callisen's operation was all but universally condemned; and numerous as they are, they might easily have been augmented. Mr. Ferguson, for example, in the paper we have already referred to, in some observations on Callisen's operation, says,

"I am inclined to think that a proposal to cut into any portion of the left side of the colon without injuring the peritoneum, either in the young or old subject, must have been made from a very limited anatomical experience; for, from what I have seen, I am positive that not one case in twenty would have admitted of an operation of the nature proposed. I make this statement as to numbers, however, merely at random, not having kept any exact account." (p. 366.)

Such was the received doctrine, such the opinion of the most respectable authorities, when M. Amussat published the series of memoirs, which, at the least, establish beyond any question, that the difficulty and danger of opening the lumbar colon, whether in the old or young subject, had been exaggerated; and further raise a strong presumption in favour of that method, as compared with Littré's. We have entered into these details because some very futile attempts have been made to deny any merit to M. Amussat in this matter, on the score that he has no claim to originality. So far as originality consists in the first rude conception of a method, coupled with an experimental failure, to render that method practicable, certainly M. Amussat has no claim to originality; but his originality, in overcoming difficulties which others failed to surmount and considered insurmountable, cannot be fairly questioned. At the same time we must add, that we consider it quite premature to assume that there is yet anything approaching to a sufficient amount of evidence to authorize us in ranking M. Amussat's operation among the recognized operations in surgery, as an operation, in fact, generally applicable in the cases in which it seems indicated; but if more extended experience speaks in its favour, the entire merit of its introduction is due to M. Amussat.

The lumbar region, in which Callisen's operation is to be performed, is a quadrilateral space, bounded above by the last false rib, below by the crest of the ilium, behind by the longissimus dorsi and sacro-lumbalis mass of muscles, and anteriorly by a vertical line falling on the centre of the crest of the ilium. The colon in this space lies above in front of the kidney, from which it is separated by fat; in the centre of the space it corresponds to the transversalis fascia, by which and a little fat it is alone separated from the quadratus lumborum muscle; below it corresponds to the crest of the ilium; anteriorly and externally it is in contact with the small intestines; its distance from the spine varies as it is contracted or distended. The important point, however, is the relation of the posterior aspect of the colon in this space to the peritoneum; is it constantly or nearly so denuded of peritoneum to a determinate extent, and that both in the adult and in the infant? M. Amussat maintains that in the adult a lumbar mesocolon never exists, that the intestine is denuded of peritoneum, at least on its posterior third, to which extent the cellular tissue, external to the peritoneum, constitutes its outer layer or sheath. This cellular space, which is formed by the separation of the layers of the peritoneum, commences at the angle of union of the transverse and lumbar colon, and has no very constant line of demarcation below, but usually terminates about the crest of the ilium. Its lateral extent is exactly defined by two of the three longitudinal muscular bands which characterize the great intestine, one running in front of the lumbar colon, the other two externally and internally, precisely along the lines whence the peritoneum is reflected on the parietes of the abdomen. But the condition of the parts varies, according to the manner in which the examination is made and with the condition of the intestine; and hence, according to M. Amussat, have arisen the misconceptions respecting the relations of the colon and the peritoneum, and the failures in attempting to open the intestine without wounding the serous membrane. If we open the abdomen in front and draw the colon forwards, the traction will sometimes cause the formation of a mesocolon; but the practically important point is that the extent of space denuded of peritoneum varies with the caliber of the intestine. When the colon is much contracted, he has seen a very small interval between the folds of the peritoneum; but as the colon dilates, for example, if we inflate it with air, the peritoneal folds and small intestines are pushed back, and the cellular space enlarges proportionally to the distension of the gut; and if the intestine is now punctured through, the air escapes, yet the colon does not retract, as the small intestines would, because it adheres, by its posterior surface, to the parietes of the abdomen; and as the same thing occurs when the colon is dilated, from tympanitis or by the feces, the operation is thus facilitated. Such being the disposition of the peritoneum, if a vertical incision along the external border of the quadratus lumborum muscle is carried too far outwards, we may, especially if the intestine is contracted, open the peritoneum, as was done by Callisen and Duret, but a transverse incision will greatly facilitate the discovery of the portion of the intestine denuded of peritoneum. The colour of the intestine sometimes offers an important peculiarity: it is generally of a greenish-yellow, longitudinally disposed, which contrasts with undulated yellow appearance of the small intestines. (First Memoir, pp. 10-17.) If, when the colon has been denuded, we open and draw the edges of the incision to the lips of the external wound, it will be found that the entire caliber of the gut will not prolapse, as would be the case with the small intestine; its posterior wall alone yields, forming a kind of prolonged tube, communicating with the intestine, so that on passing the finger into the gut there is scarcely any salient ridge (*éperon*) opposite the opening. (pp. 12-13.) The same disposition exists on both sides, but the relations of the peritoneum are more variable on the right side. In new-born infants, Sabatier and others say that the existence of a kind of lumbar mesocolon is the rule, its absence the exception, and therefore object to Callisen's operation. M. Amussat, on the contrary, maintains that the disposition of the parts in early life is even more favorable for the operation than in adults; 1st, because the situation of the lumbar colon is more constant in early life; and 2d, because the kidney forms an unerring guide to the colon, which always lies immediately external to that organ. The intestine also seems more firmly adherent to the walls of the abdomen than in adults; and as the operation could only be required in infants for congenital malformation, the gut would be distended to the maximum by the accumulation of meconium. (Third Mem. pp. 216-29.) In one dissection only out of twenty did M. Amussat find a lumbar mesocolon in the infant, and in that case the intestine was empty. (Bullet. de l'Acad. Royale de Méd., 30 Juin, 1842, p. 302.) It appears to be admitted by M. Amussat, that the operation would not be uniformly practicable on the child; as in the Third Memoir, p. 235, he says, "I am more and more satisfied that the anatomical dispositions favorable to the operation are the rule, the reverse the exception." M. Baudens, (Gazette des Hôp. 1842, No. xxvii, p. 127,) on the contrary, without disputing the absence of a lumbar mesocolon in early life, thinks that from the mere fact of the great intestine being less developed in the infant than in the adult, the cellular space in the former must be so small as to occasion great difficulty in reaching it without wounding the peritoneum, and that consequently Callisen's operation should be limited to the adult, unless indeed experience shows, which he thinks possible, that the operation in the loin, even if the peritoneum be opened, is preferable to that in the groin.

As to the mode of performing the operation, we shall advert to but a few points. M. Amussat, instead of the vertical incision mentioned by

Callisen, prefers a transverse incision, four or five fingers' breadth long, midway between the last false rib and the crest of the ilium ; and he divides the deeper parts, or even the skin if the patient is fat, crucially, in order to gain room. The advantages of the transverse incision are, 1st, that it makes the operation easier and more certain, and avoids the danger of dividing the lumbar vessels and nerves; 2, that it facilitates finding and opening the intestine without wounding the peritoneum; and 3d, it enables us to establish the artificial anus more anteriorly (First Mem., p. 24), with a view to favour which, the opening in the intestine should be drawn forward and secured to the anterior angle of the wound. (p. 20.) M. Baudens, (Loc. cit., p. 179,) however, objects to the transverse incision, the liability of wounding some of the large branches of the genito-crural and inguino-cutaneous nerves, and also that it exposes too small an extent of the intestine, which should be opened by an incision at least one inch and a half long, as otherwise the anus will contract; M. Amussat, moreover, he says, is obliged to make a crucial incision in the deep parts, which perils the lumbar arteries, is painful, and augments the extent of the wound. In order to combine the advantages of a vertical and transverse incision, without the disadvantages of either, M. Baudens proposes an oblique incision. M. Amussat warns, when operating on the infant, not to expose the kidney too much, and to avoid a crucial incision ; but in his first operation on the child, he laid bare the entire kidney, and in the second, divided both the skin and muscles crucially. (Third Memoir, pp. 229-31.)

The most delicate step of the operation is opening the intestine, the great difficulty being to identify the colon. If the intestine is contracted, it may be completely concealed by the quadratus lumborum muscle, whose external border should then be raised, or some of its fibres divided. The greenish colour of the colon may, it is said, occasionally, help us to recognize it; but pressure and percussion with the finger are the best means of ascertaining its presence; and the absence of resistance external to the great intestine is an important sign. (First Memoir, pp. 10-21.) In his third operation, M. Amussat detected the colon from the greater development of its muscular fibres compared with those of the small intestine. M. Baudens objects to all those signs. If the colon is filled with feces, it will be hard, and then may be confounded with the kidney; but if it is supple and elastic, from being distended with gas, then we cannot discriminate it by the touch from the small intestines: M. Baudens, however, boasts that he "has given this operation more certainty, a precision, so to say, mathematical," by the employment of exploratory acupuncturation ; before opening what he suspects to be the colon, he introduces an acupuncture needle furnished with a canula, and on withdrawing the needle, either gas escapes or the canula is soiled with feces if it has entered the colon. "Here, then," he exclaims, "is an infallible sign, the merit of inventing which, I trust, will not be disputed with me." (Loc. cit., p. 179.) M. Baudens only forgets to inform us how we are to know whether the instrument has penetrated the large or the small intestine. The kidney, we are told by M. Amussat, is an unerring guide to the intestine in the infant.

M. Amussat acknowledges that his operation is greatly more difficult than Littré's, (Third Memoir, p. 235;) but he elsewhere says that it is not nearly so difficult as might be imagined, being easier and much less dangerous than that for strangulated hernia for example. (First Memoir, p. 27.) Mr. Jukes, too, we find, says the

"Operation is one of easy performance, and free from those dangers which have hitherto surrounded the surgeon when applying the rules of his art to cases of obstruction in the intestinal canal, or deficiency of the anus from congenital malformation." (p. 16.)

It is essential to examine into this alleged safety and facility of the operation; and first as to its facility:

In M. Amussat's first case no difficulty occurred. In his second case, to use his own words, he was "greatly embarrassed" in detecting the colon, but at length did so, and "was relieved from a most painful uncertainty." (First Memoir, p. 61.) Again, in giving an account of his practising the operation on the dead body, he says:

"I must acknowledge that, despite all the precautions I took and the care I bestowed on this important operation, I opened the peritoneum without suspecting it. And yet I was well on my guard." (Second Mem. p. 5.)

And a little further, he observes:

"I must say that this operation is a most trying one (*extremement pénible*), because of the uncertainty and anxiety experienced by the operator........*Even when the intestine is distended* there are no sufficient means of identifying it (*rien l'indique suffisament*), although it may be perfectly laid bare; for the only sign by which we can recognize it is the elastic tumefaction, which is the same almost everywhere. The only index, the only guide, the only clue, is the kidney and the adipose cellular tissue beneath it; and when we have actually attained the cellular space on the colon, it is by the touch alone that we can recognize its thick resisting coats and muscular fibres............ At length, however, we must determine on plunging a trocar into the colon. This, I must avow, is a moment of perplexity for the operator. Instead of discouraging surgeons, I wish to inspire them with confidence; but I must also warn them of real dangers, and guard them against the *apparent facility* of this operation, when we only consider *the possibility of sometimes* performing it very quickly and easily when the intestine is greatly distended." (pp. 17-18)

In the fourth case, the anxiety, so well described in the foregoing passage, was fully experienced, and the intestine was opened "with great hesitation." And in the report of the fifth case, (Gaz. des Hôp. de Paris, 1843, p. 170,) we read:

"It would be difficult to convey an idea of the perplexity experienced by the operator and assistants, when every one now asked was the intestine seen and felt at the bottom of the wound, the colon, or the small intestine?

And again:

"The difficulty, then, is inherent in the operation itself: it must be performed without any other guide than the adipose cellular tissue of the kidney, and the touch." (Second Mem. p. 57.)

So much for *the facility and simplicity* of this operation on the adult; but we are more than once told that it is easier and more certain in the infant, because of the more constant relations of the colon, and of the guide afforded by the kidney. (Third Memoir, pp. 229-35.) In the account of the first operation on the infant, however, M. Amussat says, "After much uncertainty, *thinking* I recognized the colon, I determined to hook on the tenaculum *what I supposed* to be the gut." (p. 230 ) In the second case, the supra-renal capsule was supposed to be the colon, and was cut into, and then, after much search, an intestine presented which was *thought* to be the colon, as it really proved to be, " to the great satisfaction of all present." (pp. 231-2.) In the third case the kidney, which is to serve as a guide, was itself taken for the colon ; but a slight incision into the gland showed the mistake. The various passages we have referred to may be safely left to speak for themselves ; they sufficiently show that this operation, instead of being easy and simple, is eminently difficult and uncertain.

The alleged safety of this operation must be considered in a two-fold point of view: 1st, with respect to the risk of wounding the peritoneum ; 2d, with respect to the danger incurred when the operation is performed without injury to the serous membrane.

The risk of wounding the peritoneum must obviously, in a great measure, depend on the relative frequency of the relations between that membrane and the lumbar colon, which are described by M. Amussat as constant or nearly so ; into this purely anatomical question we shall not enter, further than stating that M. Amussat's opinions are far from being generally admitted. Many anatomists have found a lumbar mesocolon in the infant, and without going beyond the work of M. Vidal (de Cassis), whose title is prefixed to this article, we find that author stating that he has found the relations of the lumbar colon and peritoneum very variable in the adult, so that frequently, however the intestine might be distended, it could scarcely be opened without wounding the peritoneum. (pp. 113-20.) But admitting that the cellular space is always as large as M. Amussat maintains it to be, still we think it is clear that there must always be considerable danger of opening the cavity of the abdomen, when we recollect the doubt and difficulty so often experienced by M. Amussat in discriminating the colon. But is this a sufficient reason for concluding with M. Vidal that Callisen's operation should be rejected in favour of that of Littré ? Certainly not : in the former operation we run the risk of wounding that important membrane; in the latter, we are certain to do so ; and if that risk is made an objection to Callisen's operation, it is difficult to understand how the objector can gravely maintain that we should prefer Littré's operation, in which we incur the certainty.

But suppose Callisen's operation is performed without injury to the peritoneum, are we thereby ensured against the occurrence of peritoneal inflammation ? M. Amussat, Mr. Jukes, and others seem to think we are. M. Vidal argues that we are not (p. 121), and in this we perfectly agree with him. We think it probable that Amussat's second patient died of peritonitis consequent on the operation, and quite certain that Mr. Jukes's patient did so, and this is only what might be expected. Ligature of the iliac artery, lithotomy, simply leaving a catheter in the bladder, retention of urine, and a varietyof other operations, circumstances, and diseases, in which there is no wound, no primary lesion of the peritoneum, not very unfrequently excite peritonitis ; and it would be strange indeed if such an operation as we have described should not occasionally do so also ; but can any reasonable man question that whatever may be the risk of peritonitis after Callisen's operation, that risk is incalculably greater after

Littré's? In fine, then, we think both the difficulties and dangers of Callisen's operation have latterly been underrated by some writers; but, on the other hand, still more greatly exaggerated by others. M. Vidal, indeed, attempts to show from the *results of cases*, that Littré's operation has been as successful in practice as Callisen's; but he draws his conclusions from only seven cases of the former and five of the latter method: we refer our readers to the tables in this article, and leave them to form their own conclusion on this point. Whatever the result of future cases may be, the facts at present known are decidedly in favour of Callisen's or rather Amussat's operation.

The advantages of Callisen's operation, as compared with Littré's, are said to be, in addition to avoiding injury of the peritoneum, 1st. That one side of the intestine only is drawn into the wound, whence the internal salient ridge is much less marked than when an entire fold of intestine is drawn down, and consequently there is an additional chance of curing the artificial anus should it become useless. According to M. Baudens, however, this alleged advantage is imaginary. He says we can make the internal ridge sufficiently prominent to block up the entrance to the lower portion of the gut, and recommends with Bourgery that we should do so, inasmuch as the operation should only be performed in cases where we can never hope to see the feces resume their natural passage. 2d. M. Amussat maintains that the lumbar region is a much more convenient situation for an artificial anus than the iliac region; and to judge from his cases it is so, though the very reverse is alleged by M. Vidal and the opponents of Callisen's operation. 3d. It is said that there is less tendency in this operation to prolapse of the intestine. In M. Amussat's first operation on the infant, the mucous membrane of the gut prolapsed the second day, but was easily reduced. 4th. Even if the peritoneum is wounded, yet, as it is opened behind, there is less danger of fecal effusion. We may here mention, that in M. Amussat's second operation on the infant, there was hernia of the kidney in the wound on the fall of the sutures. The chief inconvenience experienced after all his successful operations was a great and incessant tendency to contraction of the artificial opening.

It has been objected to the formation of an artificial anus in the abdomen, that death is preferable to life burdened with such an infirmity. If parents on behalf of their child, or an adult on his own account, think so, it is for them to refuse their consent to the operation; but it is the duty of every surgeon to propose every resource that offers a reasonable chance of preserving life. We cannot consider in detail the indications for this operation, but shall rapidly pass in review the various circumstances in which it has been performed.

Imperforate anus is probably the case in which the propriety of forming an artificial anus in the lumbar colon will be most generally admitted, provided always that an attempt has been previously made to establish an artificial anus, *if possible*, in the anal region; nor should this attempt be lightly abandoned: we have already seen that M. Amussat, in one instance, proposed to discontinue an operation commenced in this situation; the child, it is true, died, but it will scarcely be maintained that an operation in the lumbar region would have been attended with a more favorable result. Some doubtless will agree with MM. Vidal and Baudens, that Littré's operation should be preferred, because the peritoneum is very likely to be wounded in opening the lumbar colon in the infant: be it so; we shall not rely on M. Amussat's dissections and operations to raise the contrary presumption; but as they certainly show that there is at least a chance of effecting our object, that chance should not be thrown away.

An artificial anus has also been established in the abdomen for the relief of invincible constipation, whether produced by an undiscoverable cause or known to depend on obstruction of the great intestine, either from cancerous or non-malignant disease of its own coats, or the pressure of some tumour external thereto. In a few instances also the operation has been performed when the bowels were still pervious, with the view of relieving extreme distress, caused by the difficulty of evacuating the feces, and in the hope that the progress of the organic disease by which that difficulty was caused might be retarded on removing the irritation excited by the pressure and the passage of the feces. Much difference of opinion exists as to how far the operation is indicated under the several circumstances here enumerated. We would certainly abstain from interference when the nature and situation of the obstruction were both unknown, or rather, when the situation of the obstruction could not be approximately determined by percussion, &c. We feel extremely averse to the operation in cancerous disease, but are not prepared to maintain that it could never be justifiable, for we have witnessed cases in which, though the bowels were not completely obstructed, yet the extreme misery endured by the patient would possibly tempt us, in similar circumstances, seeing the favorable result of M. Amussat's fifth case, to give the sufferer the chance of relief; and if the obstruction were complete, it would be perhaps a *duty*, rather than a matter of discretion, to attempt to prolong life. In obstruction depending on non-malignant disease, or disease not known to be malignant, there can, we conceive, be no more question as to the propriety of the operation, than there would be in a case of strangulated hernia, where the intestine was supposed or known to be sphacelated.

The situation selected for the formation of the artificial anus must obviously depend on the determination of the situation of the obstruction above which the opening must, of necessity, be made. M. Baudens maintains that, however low down in the great intestine the obstruction may be, we should always operate on the right lumbar colon, because gas, he says, is exclusively generated in the great intestine; and gas being "the scourge" of a patient with an artificial anus, we should seek to prevent its generation, by permitting the feces to traverse as short a space as possible of the large intestine. We shall not waste time in refuting this strange proposal; nor can we now enter on the very important question of the diagnosis of the nature and position of the various obstructions which may raise the question of operation, but we hope soon to have an opportunity of returning to this part of the subject.

